# *My Road Ahead* study protocol: a randomised controlled trial of an online psychological intervention for men following treatment for localised prostate cancer

**DOI:** 10.1186/1471-2407-14-83

**Published:** 2014-02-11

**Authors:** Addie C Wootten, Jo-Anne M Abbott, Katherine E Chisholm, David W Austin, Britt Klein, Marita P McCabe, Denny Meyer, Anthony J Costello, Declan G Murphy

**Affiliations:** 1 Department of Urology, Royal Melbourne Hospital, Parkville, VIC, Australia; 2 Epworth Prostate Centre, Epworth Healthcare, Richmond, VIC, Australia; 3 Australian Prostate Cancer Research, East Melbourne, VIC, Australia; 4 National eTherapy Centre, Swinburne University of Technology, Hawthorn, VIC, Australia; 5 Department of Psychology, Deakin University, Burwood, VIC, Australia; 6 DVC-R Portfolio & Faculty of Health, Federation University, Ballarat, VIC, Australia; 7 Centre for Mental Health Research, The Australian National University, Canberra, Australia; 8 Swinburne University of Technology, Hawthorn, VIC, Australia; 9 Peter MacCallum Cancer Centre, East Melbourne, VIC, Australia

**Keywords:** Prostate cancer, E-intervention, Online, Therapy, Distress, Men, Web-based, CBT

## Abstract

**Background:**

There is a need for psychosocial interventions for men with prostate cancer to promote adaptive coping with the challenges and distress associated with diagnosis, treatment and recovery. In addition, interventions are needed that help to overcome barriers to psychosocial treatment such as limited face-to-face psychosocial support services, a shortage of adequately trained professionals, geographical distance, perceived and personal stigma and a preference for consumer-centric and self-directed learning. *My Road Ahead* is an online cognitive behaviour therapy (CBT) intervention for prostate cancer. This protocol describes a randomised controlled trial (RCT) that will evaluate the efficacy of this online intervention alone, the intervention in combination with a moderated online forum, and the moderated online forum alone.

**Methods/design:**

This study utilises a RCT design with three groups receiving: 1) the 6-module My Road Ahead intervention alone; 2) the My Road Ahead intervention plus a moderated online forum; and 3) the moderated online forum alone. It is expected that 150 men with localised prostate cancer will be recruited into the RCT. Online measures will assess men’s psychological distress as well as sexual and relationship adjustment at baseline, post-intervention, 3 month follow-up and 6 month follow-up. The study is being conducted in Australia and participants will be recruited from April 2012 to Feb 2014. The primary aim of this study is to evaluate the efficacy of My Road Ahead in reducing psychological distress.

**Discussion:**

To our knowledge, My Road Ahead is the first self-directed online psychological intervention developed for men who have been treated for localised prostate cancer. The RCT will assess the efficacy of this intervention in improving psychological well-being, sexual satisfaction, relationship satisfaction and overall quality of life. If successful, this intervention could provide much needed support to men receiving treatment for localised prostate cancer in a highly accessible manner.

**Trial registration:**

Australian New Zealand Clinical Trials Registry Identifier: ACTRN12611000278932

## Background

Prostate cancer is the most commonly diagnosed non-melanoma skin cancer in males worldwide. In Australia, 19,403 men were diagnosed with prostate cancer in 2007 [1] and Australian men have a 1 in 7 chance of diagnosis of prostate cancer by age 75 and a 1 in 4 chance by age 85 [1]. Registry data demonstrates that 93% of men have localised disease at diagnosis in contemporary practice in Australia [2]. Survival rates have increased over time and 5-year prostate cancer related survival is now 92% in Australia [3]. While treatment advances have contributed to this significant improvement in survival, all treatment options will result in significant decrements in quality of life (QoL) including erectile dysfunction (ED), urinary incontinence and bowel urgency [4]. These residual symptoms can be very difficult for the patient [5] given their significant impact on the patient’s general quality of life (QoL) [6]. Survivorship issues in the prostate cancer patient population therefore pose unique challenges in terms of enhancing outcomes and reducing the negative impact on QoL.

To date, QoL research has generally focussed on the physical impact of treatments on prostate cancer patients. However, research investigating the impact of prostate cancer treatment on psychological well-being is increasing [7-12]. The results of these studies have revealed that among those who have had prostate cancer treatment, the prevalence of mood, anxiety and adjustment disorders ranges from 9-24% [8,10,13,14]. The results from other studies suggest that physical side effects of prostate cancer treatment (such as incontinence and sexual dysfunction) are especially associated with anxiety and depressive symptoms [9].

Patients also report high levels of unmet need in relation to their prostate cancer experience with one Australian study finding that 74% of men report some form of unmet need in relation to their prostate cancer diagnosis [15]. The most commonly reported area of unmet need was in the psychological support domain where 54% of men expressed that they felt some level of unmet psychological support need [15]. Sexuality was reported by 47% of men as an area where they had some level of need for assistance or support [15].

### Psychosocial interventions for men treated for localised prostate cancer

The efficacy of psychosocial support has been repeatedly reported in the general cancer setting. Unfortunately, access to evidence-based and timely psychosocial support is often limited, particularly in rural and remote regions of Australia but also in public hospitals across the country and, indeed, worldwide. It has been reported that only 14 to 21% of Australian women with cancer attend face-to-face support [16,17]. Men are lower utilisers of health services in general [18] and international reports indicate that mental health service utilisation by men is lower than women [19].

A recent systematic review of psychosocial interventions for men with prostate cancer and their partners highlighted the poor quality of current research in this field [20]. The authors concluded that group cognitive-behavioural and psycho-educational interventions with a specific focus on cancer specific distress show the most robust research evidence for efficacy, however, further research is required [20]. Another review explored psychosocial interventions addressing sexual or relationship functioning in men treated for prostate cancer [21]. This review concluded that there is some evidence to support the efficacy of psychosocial interventions in improving sexual functioning especially when delivered face-to-face or through utilisation of complex interventions that specifically target sexuality and the relationship. However, this review also concluded that further research is required [21]. Related to sexuality and intimacy, masculinity is emerging as an important area [22] and yet it is not routinely addressed and integrated into interventions.

### Online interventions

Mental and behavioural health promotion, prevention, treatment and management-oriented interventions that are delivered via the internet or other electronic technologies, with or without human support [23], often referred to as “e-Interventions”, can overcome many barriers of access that are commonly encountered in our healthcare system. Whilst the use of e-Interventions has grown in both the mental health and general health setting [24-26] it has received limited attention in the cancer setting.

In the cancer setting, most online research has focused on developing and evaluating online peer support interventions with mixed outcomes in female cancer samples [27-29]. van den Brink [30] developed an online information and support intervention for head and neck cancer patients in the Netherlands with the aim of overcoming the communication and information bottlenecks in supportive head and neck cancer care. The authors reported that patient use and satisfaction with the intervention was very high despite patients having recently undergone intensive surgical treatment. Patient age was not a barrier (mean age 59 years and range 38-78) and, despite 56% of participants having limited computer experience prior to the use of the intervention, consistently positive feedback was received. This online support intervention showed improved QoL outcomes in participants as compared to those in a control group who did not access the intervention [30]. However, the intervention focused on psycho-education and support rather than skills in coping with the experience of cancer diagnosis, treatment and recovery.

Schover et al. [31] developed and assessed the efficacy of an online sexual counselling intervention in comparison to traditional face-to-face sexual counselling for couples following treatment for localised prostate cancer. Sexual counselling involved three face-to-face sessions with a therapist while the online intervention delivered the therapy via email contact with a therapist. This study found that the delivery of the intervention via the internet was as effective as the traditional face-to-face intervention in producing enduring improvements in sexual outcomes [31]. While this study highlighted the potential utility of online interventions in this population, to date, no online self-directed intervention with minimal support and a focus on a range of identified problem areas has been developed and evaluated for localised prostate cancer.

A recent review by Leykin et al. [32] explored the role of online interventions in supporting people with cancer. This review highlighted that e-Interventions can provide the majority of people with an acceptable alternative support modality in a systematic and scalable way. Research has found that telephone and internet-based support is particularly important for men with cancer, predominately prostate cancer, living outside metropolitan regions in Australia [33]. Psycho-education and self-directed therapeutic techniques can easily be transferred to an online environment and may also break down the barriers that many people face in terms of accessing support, including lack of available resources and perceived and personal stigma, fear and uncertainty in accessing mental health services [32,34]. Leykin et al. [25] criticised current online resources for being predominately information and support based, with a need for evidenced-based treatment interventions.

The current study involves a randomised controlled trial (RCT) assessing the effectiveness of three online interventions: (i) access to an online intervention program (My Road Ahead-only); (ii) access to the online intervention plus to a moderated online forum (My Road Ahead-plus-forum); and (iii) access to the online form only (forum-only). This study design was chosen to evaluate the online intervention in comparison to a more basic level of care (some peer support) and to separate the effect of the online intervention and the peer support forum on treatment outcomes. Past RCTs have used a variety of control conditions from wait-list control groups, care as usual, or some basic information provision. Cuijpers et al. [35] have criticised past internet-based RCTs for failing to use control groups beyond a wait-list control, since there may be an overestimation of intervention efficacy when those in a wait-list control condition are unlikely to make self-directed steps to improving their situation if they are “waiting”. A web-based comparative condition has been recommended to overcome these problems [36]. Basic moderated forums have been used in previous studies as a control group as a means to control for spontaneous improvement or to control for the general attention and the possible beneficial effects from sharing one’s experience with others (e.g. [37,38]). In addition online support groups may themselves have positive effects for people with cancer (e.g., [28,29]), and the use of three groups would allow for exploration of the relative benefits of the online psychological intervention and peer support (forum).

## Method/design

### Aims and hypotheses

The overall aim of the RCT is to examine the efficacy of the online, self-directed, psychological intervention, *My Road Ahead* alone versus My Road Ahead plus use of a moderated online forum, versus the forum alone in improving participants’ psychological distress as well as sexual, relationship and psychosocial adjustment from baseline to post-intervention, 3 months and 6 months follow-up.

Primary outcome:

Psychological distress as measured by the combined total score of the DASS-21 will be used as the primary outcome measure.

It is hypothesised that:

1. Participants randomised to receive My Road Ahead, with or without access to the forum, as compared to those who receive access to the forum only condition, will demonstrate significantly greater reductions in psychological distress from pre- to post-intervention and at 3 and 6 month follow-up.

2. Participants randomised to receive access to the moderated forum in addition to My Road Ahead, as compared to those who receive access only to My Road Ahead, will demonstrate significantly greater reduction in psychological distress from pre- to post-intervention and at 3 and 6 month follow-up.

Secondary outcomes:

It is hypothesised that:

1. Participants randomised to receive My Road Ahead, with or without access to the forum, as compared to those who receive access to the forum only condition, will demonstrate significantly higher quality of life as evidenced by measures of sexual adjustment, sexual confidence and satisfaction, masculine self-esteem, marital adjustment, relationship conflict and relationship communication style from pre- to post-intervention and at 3 and 6 month follow-up.

Mediation and moderation effects:

It is hypothesised that:

1. Intervention driven reductions in psychological distress will be mediated by masculine self-esteem and moderated by sexual confidence and relationship communication style.

### Study design

This study is registered with the Australian New Zealand Clinical Trials Registry; Identifier: ACTRN12611000278932. Ethical approval to conduct the study has been obtained from Melbourne Health, Swinburne University of Technology, Deakin University and Peter MacCallum Cancer Centre Human Research Ethics Committee’s. CONSORT [39] requirements for RCTs will be adhered to throughout the study.

### Procedure

Participant consent will be obtained using an online consent process. The consent form is provided to the participant in PDF format. Participants are asked to confirm that they understand the study conditions, that they have received a copy of the participant information and that they consent to take part in the study by selecting three check-boxes during the consent process. Participants will then move on to create a personal, password protected account on the My Road Ahead website and complete the baseline questionnaires. After completing the baseline questionnaires participants will then be randomly assigned using a computer generated sequential 1-to1- to 1 allocation across the three (3) groups. Participants and researchers involved in the management of the project are not blinded to the randomisation outcomes. Group 1 participants will receive access to My Road Ahead, the 6 module online program. Group 2 participants will get access to My Road Ahead plus access to the moderated forum. Group 3 participants will receive access to the moderated forum only.

Participants in all groups will receive a weekly email reminder to return to the program and/or forum and to encourage them to continue to participate in the research over the course of the 10 weeks of the intervention phase. Participants will also receive three reminder emails to complete the questionnaires at each time point.

**Figure 1:**
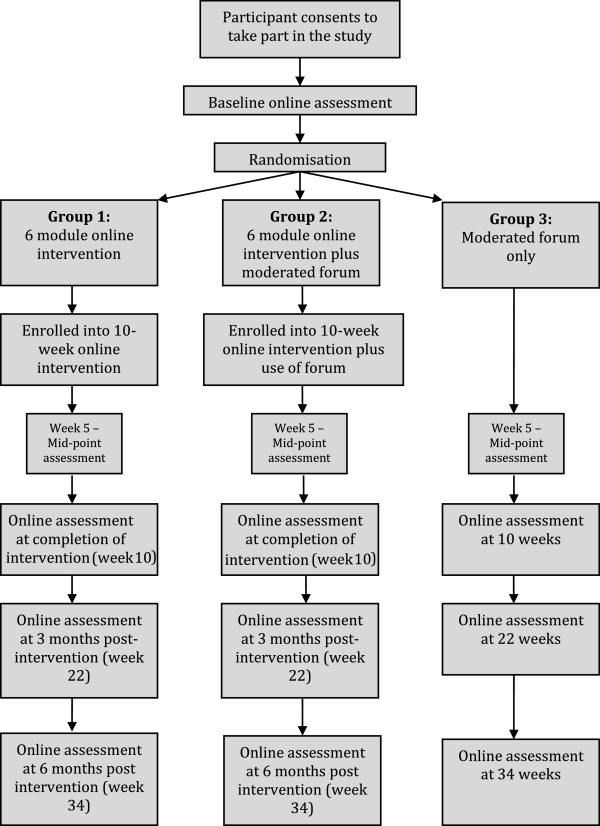
Flow chart of participant movement through RCT.

Participants in all groups will undertake the online assessment at five time points; baseline, week 5, post-intervention (week 10 for group 3) and 12 weeks post intervention (week 22 for group 3), and 6 months post-intervention (see Figure 1).

#### *Inclusion and exclusion*

Eligibility criteria include participants having been diagnosed with localised prostate cancer and have received treatment with curative intent (or currently receiving treatment) within the last 5 years. They must not have a diagnosis of advanced or metastatic disease. Participants must be able to utilise a computer or mobile device and have access to a connection to the internet at least once per week for up to one hour. They must be able to read, write and understand the English language without the assistance of an interpreter.

#### *Recruitment*

Participants will be recruited across Australia with advertisements published in various national and local newspapers as well as on various health websites (including the Prostate Cancer Foundation of Australia (http://www.prostate.org.au), beyondblue (http://www.beyondblue.org.au), MensShedOnline (http://www.theshedonline.org.au)). In addition participants will also be recruited via letters from urologists and radiation oncologists practising at the collaborating institutions including Royal Melbourne Hospital, Epworth Healthcare and Peter MacCallum Cancer Centre. Participants will be recruited across a variety of locations so as to maximise the recruitment of participants with a range of demographic variables.

#### *Randomisation*

Participant consent and sign up to the study is conducted entirely online and participants self-generate their username and password so as to ensure privacy and confidentiality. Randomisation is computer-generated on a sequential basis once the participant has completed the baseline questionnaire.

#### *My road ahead: the online psychological intervention*

My Road Ahead is a self-directed cognitive behavioural therapy (CBT) based intervention that provides psycho-education (through text, video, audio and graphics), a series of interactive exercises and regular automated feedback as participants progress through the program. The program features videos of real case experiences around coping with prostate cancer, as well as health professionals providing expert commentary.

The intervention is a six (6) module online program that offers a range of topics to work through at an individual pace across a period of ten (10) weeks. All modules are available to participants from the beginning and participants can use them in the order they chose, although a recommended sequence from 1-6 is suggested to participants.

Specific module themes are shown in Table 1.

**Table 1:** Themes of the modules within the My Road Ahead intervention

**Module**	**Themes**
1. Prostate cancer and you	• Psycho-education about common emotional responses to prostate cancer
	• Normalisation through other men’s stories
	• Interactive reflective exercises aimed at encouraging self-reflection and acknowledgement of emotional responses
2. Effective communication	• Common communication challenges in context of prostate cancer
	• Strategies to improve general and relationship communication
	• Communication enhancement exercises to complete with partner or alone
3. Physical changes	• Common physical changes following treatment for localised prostate cancer
	• Exercises to overcome anxiety and avoidance in relation to incontinence
	• Introduction to relaxation strategies
4. Sexuality and masculinity	• Education and exercises targeting negative cognitions surrounding cancer and erectile dysfunction (ED)
	• Skills training in general and sexual communication
	• Education and exercises addressing masculinity and identity issues
5. Sexuality and intimacy	• Sexual problems and cancer
	• The impact of ED and cancer on the relationship
	• Education and exercises designed to broaden definitions of sexuality and sexual behaviour to enhance sexual intimacy
	• Education about the use of medical aids for ED
6. Planning for the future	• Education and exercises related to fear and uncertainty about the future
	• Planning for the future
	• Encouragement of continued use of the skills developed from the intervention

The intervention also includes weekly offline exercises that participants download and work through. Participants have the option to do these exercises alone or with their partner; completion of exercises with the partner is self-reported by participants at the post-intervention assessment. The program also includes a weekly mood monitor that generates a graph of mood status over the course of the intervention, a log book where men can record their responses to the exercises, and a bookmarking capability so men can return to pages of particular interest.

Moderated forum:

The two moderated forum facilities are also hosted on the website, however, participants who have access to the forum in addition to the My Road Ahead program will utilise a separate forum to those participants who only have access to the forum alone, to avoid contamination across groups. Both forums are moderated by the research team and offer a ‘virtual space’ for participants to ask questions of each other. The forum content is grouped by theme and participants are encouraged to log in at least once per week and contribute to the forum. The moderator posts a comment once per week to generate conversation. Data is collected about the number and frequency of forum posts for each user and qualitative analysis of the forums will be conducted at the conclusion of the study.

### Primary outcome measure

#### *Psychological distress*

Distress will be measured using the Depression Anxiety and Stress Scales (DASS-21) short version [40]. The DASS-21 is a set of three self-report scales designed to measure the negative emotional states of depression, anxiety and stress as well as total distress [40]. Each of the three DASS-21 scales contains 7 items. The depression scale measures dysphoria, hopelessness, devaluation of life, self-deprecation, lack of interest/involvement, anhedonia and inertia. The anxiety scale measures autonomic arousal, skeletal muscle effects, situational anxiety, and subjective experience of anxious affect. The stress scale assesses difficulty relaxing, nervous arousal, being irritable and impatient. The total score is the sum of these three subscale scores. Participants are asked to rate the extent to which they have experienced each state over the past week, using a 4-point severity/frequency scale. The DASS-21 scales have good concurrent validity with the Beck measures of depression and anxiety (BDI & BAI) and good internal consistency and reliability [40].

### Secondary outcome measures

#### *The prostate cancer-related quality of life scales (PCa-QoL)*

The impact of erectile dysfunction on sexual and masculine identity will be measured using the PCa-QoL [41], which measures men’s perceptions of early prostate cancer treatment outcomes, focussing on behavioural, interpersonal and emotional changes that patients attribute to prostate cancer [41]. The scale contains 84 Likert type items that fall into 11 scales including: 1. Urinary control (behavioural and interpersonal implications of impaired control of one’s bladder); 2. Sexual ability - sexual intimacy (ability to perform sexually and feelings of frustration, embarrassment or failure); 3. Sexual confidence (confidence and anxiety about intimate activity and sexual thoughts); 4. Spouse affection (misgivings about demonstrations of affection with one’s spouse), 5. Masculine self-esteem; 6. Health worry (uncertainty about one’s health); 7. PSA concern; 8. Cancer control; 9. Informed decision; 10. Regret; and, 11. Positive outlook.

#### *International index of erectile function (IIEF)*

The IIEF [42] is a 15-item self-report measure that was developed to assess sexual function among men with Erectile Dysfunction (ED) [42]. The IIEF instructs respondents to answer items according to functioning during the past 4 weeks. Responses to items are summed to determine the total IIEF score (range 5–75). Rosen et al. [42] reported high internal consistency, for the total IIEF scale (α = 0.91), as well as high test-retest reliability (*r* = 0.82).

#### *Kansas marital satisfaction scale (KMS)*

The KMS [43] is a common measure of relationship satisfaction. It is a 3-item self-reported questionnaire measuring general relationship satisfaction. Final scores on the KMS range from 3 to 21 with higher scores indicating higher relationship satisfaction. A recent meta-analysis of 398 articles evaluated the reliability of seven different relationship satisfaction measures using Cronbach’s alpha estimates [44]. The authors found the KMS to be the strongest overall measure based on reliability alone with an average of *r* = .95 across studies.

#### *Communication patterns questionnaire-short form (CPQ-SF)*

The CPQ-SF [45,46] is an 11-item self-report questionnaire that measures communication within intimate relationships. The CPQ-SF asks respondents to identify typical communication styles when either issues or problems arise and during discussion of issues or problems. Respondents are asked to rate the likelihood that certain issues/problems occur on a 9-point scale from very unlikely to very likely.

#### *Dyadic sexual communication scale (DSC)*

The DSC [47] was originally developed as a 13-item scale to measure a respondent’s self-reported perception of their communication with their partner related to sexual relationships. Subsequently, Choi, Catania, and Dolcini [48] created a 4-item version of the DSC scale. The short-form DSC has 4 items requesting ratings on a 5-point scale: strongly disagree, disagree, neutral, agree, and strongly agree. Reliability of the 4 item version of the DSC was found to be acceptable taken from a male sample as part of the larger study mentioned above (Cronbach’s alpha = .65) [48].

#### *General confidence*

Because of the strong link between sexual potency and general confidence, a 1-item scale was created to assess general confidence (*I feel confident in most areas of my life*) which will be rated a 5-point scale: *almost never/never* to *almost always/always*.

#### *Healthcare service utilisation and demographics*

Participants will also be asked to record the number of contacts they have with additional support services including psychiatrists, psychologists and social workers as well as attending a support group or accessing support from the cancer council or other online resources. Degree of engagement of the participant’s partner in the program and completion of the exercises will also be assessed post-intervention.

Demographic details include age, date of birth, marital status, employment status, gross annual income, ethnicity, languages spoken. Other participant details including postcode, prostate cancer date of diagnosis, date of treatment, type of treatment, use of oral erectile function medications (e.g. use of Viagra or cialis), use of injection therapies for erectile function (e.g. Caverject injections), and use of other mechanical aids for erectile function will also be collected.

#### *Program and forum satisfaction questionnaires*

Program completion will be tracked and recorded through the online system which will allow reporting of the proportion of the program completed by each user as well as details about which modules are most accessed. A questionnaire has been developed for this study for the purpose of measuring the level of program satisfaction and level of inclusion of partners. Respondents will be presented with open-ended questions relating to the “best” and “worst” parts of the program and how the program could be improved, including any additional features they might find useful. If men do not work through all the modules they will be asked for reasons. An equivalent questionnaire has been developed to measure satisfaction with the moderated forum.

### Participants

A medium effect size is estimated for the primary outcome measure, total distress, as measured by the DASS-21 total score between groups across the five assessment points. To power the study at the 80% level, based on an alpha level of .05 with an estimated medium effect size (f = .25) and with three groups completing five assessment points, 125 complete responses will be required. To account for an estimated 20% drop-out rate a total of 150 participants will be required; 50 per group.

### Statistical analyses

Data will be analysed using repeated measures Multivariate Analysis of Variance (MANOVA) with between and within subjects factors examined while controlling for age. To examine any bias that may arise as a consequence of attrition, baseline data will be examined to explore if there are any statistically significant factors associated with participants who drop out compared to those who do not. This will allow an analysis for attrition bias in the final results based on the predicted probability of attrition as recommended by Rubin [49] and Heckman [50]. An intention to treat analysis and then longitudinal hierarchical linear model analyses will be performed [51,52], avoiding the need for the imputation of missing data.

## Discussion

To our knowledge, My Road Ahead is the first self-directed online psychological intervention developed for localised prostate cancer. The RCT will assess the efficacy of this intervention in improving psychological well-being, sexual satisfaction, relationship satisfaction and overall quality of life. If successful this intervention could provide much needed support to men receiving treatment for localised prostate cancer in an accessible, appealing and accessible manner.

This study aims to evaluate the effectiveness of the developed intervention by comparing the outcomes of participants who receive access to My Road Ahead, with or without access to a forum, to the outcomes of participants who receive access to a forum only. This design enables evaluation of the benefits of the peer discussion forum in addition to the developed intervention. This novel approach to evaluation of the contribution of the peer discussion forum will provide much needed data about the role of psychological strategies and peer discussion.

Lack of available psychosocial support services for men treated for prostate cancer is a significant problem worldwide. Integration of psychosocial services within the treatment team is very rare and patient reported unmet needs in this domain are very high. If My Road Ahead is successful we hope to be able to provide access to the program to all men treated for localised prostate cancer in Australia thereby increasing access by reducing barriers to access as well as reducing stigma associated with accessing psychosocial services.

## Competing interests

The authors declare they have no competing interests.

## Authors’ contributions

ACW – 25% contribution to paper including study design, clinical content development, data analysis and report writing. JMA – 15% contribution to paper including study design, clinical content development, data analysis and report writing. KC – 12% contribution to paper including study design, clinical content development, data analysis and report writing. DA – 10% contribution to paper including study design and report writing. BK – 10% contribution to paper including study design and report writing. MM – 10% contribution to paper including study design and report writing. DM - 8% contribution to paper including power calculations, statistical analysis, and report writing. AJC – 5% contribution to paper including participant recruitment and report writing. DGM – 5% contribution to paper including participant recruitment and report writing. All authors read and approved the final manuscript.

## Author’s information

ACW is a clinical psychologist from the Royal Melbourne Hospital, the director of clinical and allied health research at the Epworth Prostate Centre and the eHealth Research Manager at Australian Prostate Cancer Research. JMA is a health psychologist and research fellow at the National eTherapy Centre, Swinburne University of Technology. KC is a registered psychologist at Deakin University. DA is an associate professor in psychology at Deakin University with extensive experience in the e-therapy field. BK is a professor of psychology from Federation University, a Visiting Fellow at the ANU and an Adjunct Professor at Swinburne University of Technology, with extensive experience in the e-therapy field. MM is a professor of Psychology at Deakin University. DM is a professor of statistics at Swinburne University. AJC if a professor of Urology at Royal Melbourne Hospital and the head of the department of Urology. DGM is an associate professor of urology and is the director of robotic surgery at Peter MacCallum Cancer Centre.

## Pre-publication history

The pre-publication history for this paper can be accessed here:

http://www.biomedcentral.com/1471-2407/14/83/prepub

## References

[B1] AIHW Cancer in Australia 2010: an overviewvol. Cancer series no. 60 2010 Canberra: AIHW

[B2] Evans SM Millar JL Davis ID Murphy DG Bolton DM Giles GG Frydenberg M Andrianopoulos N Wood JM Frauman AG Patterns of care for men diagnosed with prostate cancer in Victoria from 2008 to 2011 Med J Aust 2013 198 10 540 545 10.5694/mja12.11241 23725268

[B3] AIHW & AACR Cancer in Australia 2012: an overviewvol. Cancer Series no. 74 2012 Canberra: AIHW

[B4] Resnick MJ Koyama T Fan KH Albertsen PC Goodman M Hamilton AS Hoffman RM Potosky AL Stanford JL Stroup AM Long-term functional outcomes after treatment for localized prostate cancer N Engl J Med 2013 368 5 436 445 10.1056/NEJMoa1209978 23363497 PMC3742365

[B5] Gray RE Fitch MI Phillips C Labrecque M Klotz L Presurgery experiences of prostate cancer patients and their spouses Cancer Pract 1999 7 130 135 10.1046/j.1523-5394.1999.07308.x 10352075

[B6] Bokhour BG Clark JA Inui TS Silliman RA Talcott JA Sexuality after treatment for early prostate cancer: Exploring the meanings of erectile dysfunction J Gen Intern Med 2001 16 649 655 10.1111/j.1525-1497.2001.00832.x 11679031 PMC1495277

[B7] Blank TO Bellizzi KM After prostate cancer: predictors of well-being among long-term prostate cancer survivors Cancer 2006 106 10 2128 2135 10.1002/cncr.21865 16607648

[B8] Couper JW Bloch S Love A Duchesne G Macvean M Kissane DW The psychosocial impact of prostate cancer on patients and their partners Med J Aust 2006 185 8 428 432 17137432 10.5694/j.1326-5377.2006.tb00640.x

[B9] Eller LS Lev EL Gejerman G Colella J Esposito M Lanteri V Scheuch J Munver R Lane P Junchaya C Prospective study of quality of life of patients receiving treatment for prostate cancer Nurs Res 2006 55 2 Suppl S28 S36 16601630

[B10] Hervouet S Savard J Simard S Ivers H Laverdiere J Vigneault E Fradet Y Lacombe L Psychological functioning associated with prostate cancer: cross-sectional comparison of patients treated with radiotherapy, brachytherapy, or surgery J Pain Symptom Manage 2005 30 5 474 484 10.1016/j.jpainsymman.2005.05.011 16310621

[B11] Korfage IJ Essink-Bot ML Janssens AC Schroder FH de Koning HJ Anxiety and depression after prostate cancer diagnosis and treatment: 5-year follow-up Br J Cancer 2006 94 8 1093 1098 10.1038/sj.bjc.6603057 16622434 PMC2361242

[B12] Wootten AC Burney S Foroudi F Frydenberg M Coleman G Ng KT Psychological adjustment of survivors of localised prostate cancer: investigating the role of dyadic adjustment, cognitive appraisal and coping style Psychooncology 2007 16 11 994 1002 10.1002/pon.1159 17278153

[B13] Frick E Tyroller M Panzer M Anxiety, depression and quality of life of cancer patients undergoing radiation therapy: A cross sectional study in a community hospital outpatient centre Eur J Cancer Care 2007 16 130 136 10.1111/j.1365-2354.2006.00720.x 17371421

[B14] Pirl WF Siegel GI Goode MJ Smith MR Depression in men receiving androgen deprivation therapy for prostate cancer: a pilot study Psychooncology 2002 11 6 518 523 10.1002/pon.592 12476433

[B15] Smith DP Supramaniam R King MT Ward J Berry M Armstrong BK Age, health, and education determine supportive care needs of men younger than 70 years with prostate cancer J Clin Oncol 2007 25 18 2560 2566 10.1200/JCO.2006.09.8046 17577034

[B16] Wade TD Lee C The impact of breast cancer on the lives of middle-aged women: results from the Australian longitudinal study of women's health Health Psychol 2005 24 3 246 251 15898859 10.1037/0278-6133.24.3.246

[B17] Winefield HR Coventry BJ Lewis M Harvey EJ Attitudes of patients with breast cancer toward support groups J Psychosoc Oncol 2003 21 2 39 54 10.1300/J077v21n02_03

[B18] Commonwealth Department of Health and Aged Care Insights into the utilisation of health services in Australia based on linked administrative data Occasional Papers 2000 Canberra: Commonwealth Department of Health and Aged Care

[B19] Earle CC Neville BA Fletcher R Mental health service utilization among long-term cancer survivors J Cancer Surviv 2007 1 2 156 160 10.1007/s11764-007-0013-2 18648956

[B20] Chambers SK Pinnock C Lepore SJ Hughes S O'Connell DL A systematic review of psychosocial interventions for men with prostate cancer and their partners Patient Educ Couns 2011 85 2 e75 e88 10.1016/j.pec.2011.01.027 21334159

[B21] Chisholm KE McCabe MP Wootten AC Abbott JM Review: psychosocial interventions addressing sexual or relationship functioning in men with prostate cancer J Sex Med 2012 9 5 1246 1260 10.1111/j.1743-6109.2012.02687.x 22458946

[B22] Zaider T Manne S Nelson C Mulhall J Kissane D Loss of masculine identity, marital affection, and sexual bother in men with localized prostate cancer J Sex Med 2012 9 10 2724 2732 10.1111/j.1743-6109.2012.02897.x 22989267 PMC5180593

[B23] Klein B e-Interventions and psychology: Time to log on! InPsych 2010 32 20 22

[B24] Spek V Cuijpers P Nyklicek I Riper H Keyzer J Pop V Internet-based cognitive behaviour therapy for symptoms of depression and anxiety: a meta-analysis Psychol Med 2007 37 3 319 328 10.1017/S0033291706008944 17112400

[B25] Ritterband LM Thorndike FP Gonder-Frederick LA Magee JC Bailey ET Saylor DK Morin CM Efficacy of an Internet-based behavioral intervention for adults with insomnia Arch Gen Psychiatry 2009 66 7 692 698 10.1001/archgenpsychiatry.2009.66 19581560 PMC3723339

[B26] Ritterband LM Ardalan K Thorndike FP Magee JC Saylor DK Cox DJ Sutphen JL Borowitz SM Real world use of an Internet intervention for pediatric encopresis J Med Internet Res 2008 10 2 e16 10.2196/jmir.1081 18653440 PMC2483922

[B27] Salzer MS Palmer SC Kaplan K Brusilovskiy E Ten Have T Hampshire M Metz J Coyne JC A randomized, controlled study of Internet peer-to-peer interactions among women newly diagnosed with breast cancer Psychooncology 2010 19 4 441 446 10.1002/pon.1586 19484712

[B28] Wiljer D Urowitz S Barbera L Chivers ML Quartey NK Ferguson SE To M Classen CC A qualitative study of an internet-based support group for women with sexual distress due to gynecologic cancer J Cancer Educ 2011 26 3 451 458 10.1007/s13187-011-0215-1 21594587

[B29] Winzelberg AJ Classen C Alpers GW Roberts H Koopman C Adams RE Ernst H Dev P Taylor CB Evaluation of an internet support group for women with primary breast cancer Cancer 2003 97 5 1164 1173 10.1002/cncr.11174 12599221

[B30] van den Brink JL Moorman PW de Boer MF van Bemmel JH Pruyn JF Verwoerd CD An information system to support the care for head and neck cancer patients Support Care Cancer 2003 11 7 452 459 10.1007/s00520-002-0425-5 12707835

[B31] Schover LR Canada AL Yuan Y Sui D Neese L Jenkins R Rhodes MM A randomized trial of internet-based versus traditional sexual counseling for couples after localized prostate cancer treatment Cancer 2012 118 2 500 509 10.1002/cncr.26308 21953578 PMC4022136

[B32] Leykin Y Thekdi SM Shumay DM Munoz RF Riba M Dunn LB Internet interventions for improving psychological well-being in psycho-oncology: review and recommendations Psychooncology 2012 21 9 1016 1025 10.1002/pon.1993 21608075 PMC3181007

[B33] Corboy D McLaren S McDonald J Predictors of support service use by rural and regional men with cancer Aust J Rural Health 2011 19 4 185 190 10.1111/j.1440-1584.2011.01210.x 21771159

[B34] Schulze B Mental-health stigma: expanding the focus, joining forces Lancet 2009 373 9661 362 363 10.1016/S0140-6736(08)61818-8 19162313

[B35] Cuijpers P van Straten A Warmerdam L Andersson G Psychological treatment of depression: a meta-analytic database of randomized studies BMC Psychiatry 2008 8 36 10.1186/1471-244X-8-36 18485191 PMC2408566

[B36] Danaher BG Seeley JR Methodological issues in research on web-based behavioral interventions Ann Behav Med 2009 38 1 28 39 10.1007/s12160-009-9129-0 19806416 PMC3846298

[B37] Andersson E Walen C Hallberg J Paxling B Dahlin M Almlov J Kallstrom R Wijma K Carlbring P Andersson G A randomized controlled trial of guided Internet-delivered cognitive behavioral therapy for erectile dysfunction J Sex Med 2011 8 10 2800 2809 10.1111/j.1743-6109.2011.02391.x 21797983

[B38] Hesser H Gustafsson T Lunden C Henrikson O Fattahi K Johnsson E Zetterqvist Westin V Carlbring P Maki-Torkko E Kaldo V A randomized controlled trial of Internet-delivered cognitive behavior therapy and acceptance and commitment therapy in the treatment of tinnitus J Consult Clin Psychol 2012 80 4 649 661 22250855 10.1037/a0027021

[B39] Altman DG Schulz KF Moher D Egger M Davidoff F Elbourne D Gotzsche PC Lang T The revised CONSORT statement for reporting randomized trials: explanation and elaboration Ann Intern Med 2001 134 8 663 694 10.7326/0003-4819-134-8-200104170-00012 11304107

[B40] Lovibond PF Lovibond SH The structure of negative emotional states: comparison of the depression anxiety stress scales (DASS) with the beck depression and anxiety inventories Behav Res Ther 1995 33 3 335 343 10.1016/0005-7967(94)00075-U 7726811

[B41] Clark JA Bokhour BG Inui TS Silliman RA Talcott JA Measuring patients' perceptions of the outcomes of treatment for early prostate cancer Med Care 2003 41 8 923 936 10.1097/00005650-200308000-00006 12886172

[B42] Rosen RC Riley A Wagner G Osterloh IH Kirkpatrick J Mishra A The international index of erectile function (IIEF): a multidimensional scale for assessment of erectile dysfunction Urology 1997 49 6 822 830 10.1016/S0090-4295(97)00238-0 9187685

[B43] Schumm WR Anderson SA Benigas JE McCutchen MB Griffin CL Morris JE Race GS Criterion-related validity of the Kansas Marital Satisfaction Scale Psychol Rep 1985 56 3 718 10.2466/pr0.1985.56.3.718 4034824

[B44] Graham JM Diebels KJ Barnow ZB The reliability of relationship satisfaction: a reliability generalization meta-analysis J Fam Psychol 2011 25 1 39 48 21355645 10.1037/a0022441

[B45] Christensen A Heavey CL Gender and social structure in the demand/withdraw pattern of marital conflict J Pers Soc Psychol 1990 59 1 73 81 2213491 10.1037//0022-3514.59.1.73

[B46] Futris TG Campbell K Nielsen RB Burwell ST The Communication patterns questionnaire-short form: a review and assessment Fam J 2010 18 275 287 10.1177/1066480710370758

[B47] Catania JA Davis CM, Yarber WL, Bauserman R, Schreer G, Davis SL Dyadic Sexual Communication Scale Handbook of sexuality-related measures 1998 London: Sage 129 131

[B48] Choi KH Catania JA Dolcini MM Extramarital sex and HIV risk behavior among US adults: results from the National AIDS behavioral survey Am J Public Health 1994 84 12 2003 2007 10.2105/AJPH.84.12.2003 7998648 PMC1615405

[B49] Rubin DB Estimating causal effects from large data sets using propensity scores Ann Intern Med 1997 127 8 Pt 2 757 763 9382394 10.7326/0003-4819-127-8_part_2-199710151-00064

[B50] Heckman JJ Sample selection bias as a specification error Econometrica 1979 47 1 153 161 10.2307/1912352

[B51] Heck RH Thomas SL Tabata LN An Introduction to Multilevel Modeling Techniques (Quantitative Methodology Series) 2008 Mahwah, NJ: Lawrence Erlbaum

[B52] Raudenbush SW Bryk AS Hierarchical Linear Models: Applications and Data Analysis Methods 2008 Thousand Oaks, CA: Sage

